# Artificial intelligence technology in ophthalmology public health: current applications and future directions

**DOI:** 10.3389/fcell.2025.1576465

**Published:** 2025-04-17

**Authors:** ShuYuan Chen, Wen Bai

**Affiliations:** ^1^ Xuzhou Medical University, Xuzhou, China; ^2^ The Affiliated Eye Hospital, Nanjing Medical University, Nanjing, China; ^3^ The Fourth School of Clinical Medicine, Nanjing Medical University, Nanjing, China

**Keywords:** ophthalmology, public health, artificial intelligence, digital health, telemedicine

## Abstract

Global eye health has become a critical public health challenge, with the prevalence of blindness and visual impairment expected to rise significantly in the coming decades. Traditional ophthalmic public health systems face numerous obstacles, including the uneven distribution of medical resources, insufficient training for primary healthcare workers, and limited public awareness of eye health. Addressing these challenges requires urgent, innovative solutions. Artificial intelligence (AI) has demonstrated substantial potential in enhancing ophthalmic public health across various domains. AI offers significant improvements in ophthalmic data management, disease screening and monitoring, risk prediction and early warning systems, medical resource allocation, and health education and patient management. These advancements substantially improve the quality and efficiency of healthcare, particularly in preventing and treating prevalent eye conditions such as cataracts, diabetic retinopathy, glaucoma, and myopia. Additionally, telemedicine and mobile applications have expanded access to healthcare services and enhanced the capabilities of primary healthcare providers. However, there are challenges in integrating AI into ophthalmic public health. Key issues include interoperability with electronic health records (EHR), data security and privacy, data quality and bias, algorithm transparency, and ethical and regulatory frameworks. Heterogeneous data formats and the lack of standardized metadata hinder seamless integration, while privacy risks necessitate advanced techniques such as anonymization. Data biases, stemming from racial or geographic disparities, and the “black box” nature of AI models, limit reliability and clinical trust. Ethical issues, such as ensuring accountability for AI-driven decisions and balancing innovation with patient safety, further complicate implementation. The future of ophthalmic public health lies in overcoming these barriers to fully harness the potential of AI, ensuring that advancements in technology translate into tangible benefits for patients worldwide.

## 1 Introduction

Global eye health has become a major public health challenge. In 2020, around 1.1 billion people worldwide were affected by visual impairment, including 43 million blind individuals, 295 million with moderate to severe visual impairment, 258 million with mild visual impairment, and 510 million with myopia (Blindness et al., 2021b; Burton et al., 2021; Philippin et al., 2024). By 2050, the number of blind people is expected to increase by 610 million, and 474 million people will face moderate or severe visual impairment (Blindness et al., 2021b). This burden disproportionately affects developing nations and aging populations, with 80% of cases concentrated in these vulnerable communities (Yusufu et al., 2021). This growing issue affects not only individual health but also public health systems, leading to reduced quality of life, higher economic costs, lower productivity, and increased social welfare expenses. Notably, visual impairment among the working-age population led to a staggering $410.7 billion in global productivity losses in 2020 (Blindness et al., 2021a). Even presbyopia alone, assuming working individuals under 65, could result in $25.367 billion in losses, representing 0.037% of global GDP (Frick et al., 2015). These figures underscore the urgent and imperative need for concerted global action to address eye health challenges.

The traditional ophthalmic public health system faces several challenges, primarily the uneven distribution of medical resources. Low-income countries have just 3.7 ophthalmologists per million people, while high-income countries have 76.2 (Resnikoff et al., 2020). This 20 times gap has led to the desert of eye disease treatment in poor areas, as seen in the northern states of Nigeria, where 92% of children with blindness lack surgical intervention (Resnikoff et al., 2020). In low- and middle-income countries, limited resources worsen eye health issues, resulting in more untreated vision impairments (Burton et al., 2021). High-income countries, despite stronger healthcare systems, face new challenges due to changing patterns of eye diseases (Ameenat Lola Solebo et al., 2022). As infectious eye diseases decrease, chronic conditions rise (Wong et al., 2014; Goodman et al., 2023). Additionally, many healthcare workers lack proper training to diagnose and treat common eye diseases (Freeman et al., 2013). Public awareness of eye health is low, leading to delayed treatment and irreversible vision loss. The annual economic loss of productivity loss is more than 411 billion US dollars, mainly from preventable conditions such as uncorrected presbyopia (Capo et al., 2022; Williams and Sahel, 2022). These challenges not only threaten global eye health but also hinder social and economic progress, making the need for effective solutions urgent.

The rapid development of AI offers valuable solutions to challenges in ophthalmic public health. AI can efficiently screen for eye diseases and diagnose them early in large populations (Hamet and Tremblay, 2017). It also helps optimize medical resource allocation, with AI-assisted telemedicine (Li J. O. et al., 2021; Simon et al., 2024) and mobile apps (Suo et al., 2022; Pundlik et al., 2023) providing high-quality diagnostic support to primary healthcare facilities. In research, ophthalmic AI can analyze large amounts of medical data, helping researchers identify new disease patterns and treatment methods (Ting et al., 2019). Machine learning also aids in discovering biomarkers, supporting early diagnosis and personalized treatments for eye diseases (Linde et al., 2024).

This review examines AI applications in ophthalmic public health, including data governance, disease screening, risk prognostication, healthcare optimization, and patient management. It is relevant to stakeholders such as ophthalmic patients, primary healthcare providers, and ophthalmologists. By synthesizing research and case studies, the review highlights AI’s clinical potential and implementation challenges, offering insights into its future integration in ophthalmic public health systems.

## 2 Application of AI models and algorithms in the field of ophthalmology public health

### 2.1 Informatization of ophthalmology

**FIGURE 1:**
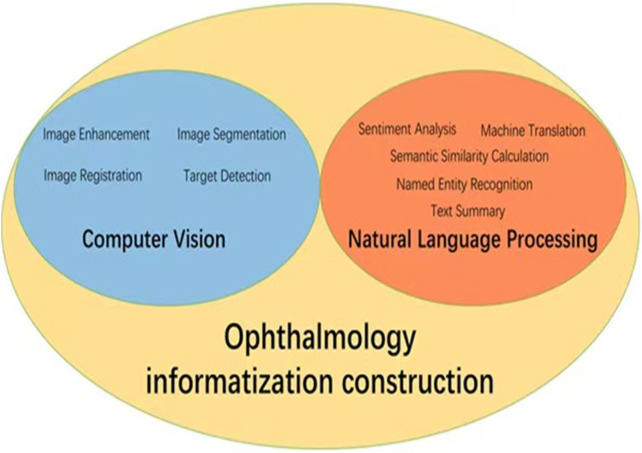
Application diagram of AI in ophthalmology informatization construction. The core AI technologies in ophthalmic informatics focus on computer vision methodologies (encompassing image enhancement, segmentation, registration and target detection) coupled with natural language processing implementations (sentiment analysis, text summary, machine translation, named entity recognition, and semantic similarity calculation).

With the rapid development of information technology, AI is becoming increasingly important in healthcare, especially in ophthalmic public health. Data informatization is key to improving healthcare efficiency and quality. AI supports clinical diagnosis, research, and public health decisions by efficiently processing ophthalmic data. Computer vision and natural language processing are crucial for ophthalmology informatization. Relevant algorithms are shown in Figure 1.

Computer vision is essential in medical diagnosis by analyzing and interpreting images, enabling computers to process medical images like fundus photos, corneal topography, and OCT images (Esteva et al., 2021). This helps advance the informatization of ophthalmology, with key algorithms such as image enhancement (Shen et al., 2021; Wang J. et al., 2021), segmentation (Li X. et al., 2021; Xing et al., 2022), target detection (Son et al., 2020; Dai et al., 2021), and image registration (Wang Y. et al., 2021; Zhang et al., 2022). For instance, image enhancement improves low-quality fundus photos. Shen et al. developed a method using image decomposition and visual adaptation to enhance clarity and contrast (Shen et al., 2021). Lee et al. introduced a generative adversarial network (GAN) approach, termed FQ-UWF (Lee et al., 2024), that enhances low-quality wide-angle fundus images unsupervised, providing clearer views for more accurate diagnoses. Image segmentation divides medical images into key regions for diagnosis. Li et al. proposed a semi-supervised model that improves accuracy by combining unlabeled and limited annotated data (Li X. et al., 2021). Target detection, using deep learning, identifies specific areas in images. Son et al. developed models for detecting retinal abnormalities in fundus images, enabling quick and accurate identification of lesions like microaneurysms and bleeding (Son et al., 2020). Image registration aligns images from different times or modes for comparison. Wang introduced a multimodal retinal image registration method using weakly supervised deep learning (Wang Y. et al., 2021), while Zhang proposed a two-step method for optimizing image alignment (Zhang et al., 2022). A summary of these computer vision algorithms applied to ophthalmic images is shown in Table 1.

**TABLE 1:** Summary of AI algorithms in computer vision.

No.	Algorithm/Model	Application	References
1	GAN (Generate Countermeasure Network)	Ultra-wide angle fundus image quality enhancement improves the clarity and contrast of peripheral retinal microlesions in diabetic retinopathy patients.	Lee et al. (2024)
2	RNN (Recurrent Neural Network)	Analysis of choroidal neovascularization activity using OCT longitudinal sequences.	Romo-Bucheli et al. (2020)
3	Mbsanet (CNN + SA mechanism)	Combined recognition of multiple fundus diseases enables the detection of hard exudates and glaucomatous optic disc depression in diabetic retinopathy.	Wang et al. (2023a)
4	Canny edge detection	Quantitative analysis of retinal vascular morphology detects vascular tortuosity and branch abnormalities in diabetic retinopathy patients.	Li and Chutatape (2004)
5	Hybrid 2D-3D CNN	Three-dimensional registration of OCT layered structure enables longitudinal tracking of retinal thickness changes in diabetic macular edema patients.	Khansari et al. (2020), Liu et al. (2024a)
6	U-Net and MSAG network	Quantitative choroidal volume measurement assesses posterior scleral staphyloma in high myopia patients.	Tsuji et al. (2020), Kundu et al. (2022), Xu et al. (2024)
7	CoFe net (Fundus enhancement network)	Low-quality fundus image enhancement improves the visibility of microaneurysms and bleeding points in diabetic retinopathy patients.	Shen et al. (2021)
8	Image decomposition and visual adaptation	Enhancing the contrast of vascular stenosis, arteriovenous crossing signs, and bleeding areas in hypertensive retinopathy.	Wang et al. (2021a)
9	Transformation consistency self-integration model	Semi-supervised glaucoma optic disc/cup segmentation reduces label dependence, improves cup-to-disc ratio calculation accuracy	Li et al. (2021b)
10	Weakly supervised deep learning framework	The fusion of fundus color photography and fluorescein angiography (FA) enables precise localization of non-perfusion areas in diabetic retinopathy	Wang et al. (2021b)
11	Two-step registration method	Efficiently register OCT and OCTA images to evaluate choroidal neovascularization (CNV) morphology and blood flow changes in the macular region.	Zhang et al. (2022)
12	Unpaired generation model	Ultra-widefield (UWF) image quality enhancement improves peripheral retinal imaging, increases the detection rate of retinal holes and lattice degeneration	Lee et al. (2024)

Natural language processing (NLP) focuses on processing and analyzing text data, enabling the extraction and understanding of information through automated methods (Wu et al., 2022). It supports medical decision-making and management by analyzing electronic medical records, research literature, patient questionnaires, and other textual data. NLP enhances clinical decision-making, patient monitoring, educational consultations, medical record management, and accelerates scientific research. Key related algorithms include sentiment analysis (Nguyen et al., 2021), text classification (Zhao et al., 2025), text summarization (Chotcomwongse et al., 2024), named entity recognition (NER) (Macri et al., 2023), and semantic similarity calculation (Bible et al., 2017). For example, text classification can analyze electronic medical records to categorize diseases, creating algorithms that help doctors quickly access relevant information, improving efficiency (Stein et al., 2019). NER technology extracts crucial entities such as disease names, treatment plans, and drug dosages from medical records, aiding clinical decision-making and research (Hossain et al., 2023). Maganti et al. demonstrated NER’s use in microbial keratitis analysis, improving diagnosis and treatment by extracting data like inflammation levels and pathogen types from clinical notes (Wu et al., 2022). Machine translation technology enables the translation of medical records and documents across languages, facilitating cross-language communication and collaboration. Semantic similarity computation finds similarities between medical records and literature, helping identify disease patterns and treatment outcomes (Mulyar et al., 2021). For instance, Yang et al. analyzed medical records from diabetic retinopathy patients to identify treatment effectiveness across different groups, aiding doctors in optimizing treatment strategies (Yang et al., 2021). A summary of these NLP algorithms in ophthalmology is provided in Table 2.

**TABLE 2:** Summary of AI algorithms in natural language processing.

No.	Algorithm/Model	Application	References
1	DPC code selection algorithm	Uses text mining to extract key info from discharge summaries and auto-select DPC codes, improving record standardization and accuracy	Suzuki et al. (2008)
2	Clinical model clustering (semantic similarity)	Uses NLP to cluster models based on semantic similarity, aiding personalized treatment by identifying patient group characteristics	Goeg et al. (2015)
3	Information extraction algorithm	Uses NLP to improve case detection sensitivity and specificity by extracting information from electronic records	Ford et al. (2016)
4	De-identification method	Uses NLP to quickly de-identify records, ensuring privacy during sharing and analysis, suitable for large-scale data	Wellner et al. (2007)
5	Algorithm evaluation	Evaluates algorithm performance in identifying ophthalmic conditions by analyzing structured and unstructured data to improve accuracy	Stein et al. (2019)
6	NLP (Natural Language Processing)	Reviews NLP applications in ophthalmology, including clinical document analysis, image report generation, and patient communication	Wu et al. (2022)
7	Deep learning + NLP	Introduces deep learning combined with NLP for ophthalmology, covering disease diagnosis, treatment recommendations, and patient history summaries	Yang et al. (2021)

In the future, as technology improves and data grows, AI will play a bigger role in ophthalmic data management. These technologies have the potential to simplify medical processes, but careful attention must be paid to data privacy, algorithm bias, and the need for ongoing training of healthcare professionals to ensure effective and equitable implementation.

### 2.2 Ophthalmic disease screening and monitoring

#### 2.2.1 Early screening tool

Early screening is essential for preventing and controlling eye diseases. Traditional methods, such as door-to-door screening by field workers, rely heavily on the expertise of ophthalmologists but face challenges like high costs and low efficiency (Vujosevic et al., 2020). AI-enabled screening tools can enhance this approach by assisting in the identification and referral of patients who need further intervention. This enables large-scale, efficient screening. Many reviews have highlighted AI’s role in screening ophthalmic diseases like diabetic retinopathy (Teixeira et al., 2024), glaucoma (Vieira et al., 2025), and macular degeneration (Kang et al., 2024).

The core of early screening tools lies in image recognition technology, which can automatically analyze and diagnose medical images such as fundus images and optical coherence tomography (OCT) scans. Common deep learning models used in image recognition include convolutional neural networks (CNN) (Schmidt-Erfurth et al., 2018; Liu et al., 2020), generative adversarial networks (GAN) (Barrera et al., 2023), and attention mechanisms (Bera et al., 2021). Among these, CNNs are the most widely used in ophthalmic image recognition due to their strong ability to extract and classify image features. By training on large ophthalmic image datasets, CNNs can learn the characteristics of various eye diseases and accurately diagnose them. Google’s DeepMind team has developed a CNN-based eye disease screening system that detects various eye diseases in fundus images with accuracy similar to professional ophthalmologists (Li Y. et al., 2022). The system extracts high-dimensional features from images through multi-level convolution layer and pooling layer, and finally classifies them through full connection layer. The results show that this method performs well in the recognition of multiple ophthalmic diseases, with ACC and AUC reaching 97.05% and 98.66% (Li Y. et al., 2022). Deep learning also handles complex multimodal data. Multimodal learning models, such as attention-based fusion networks, can extract key features from various data sources, improving diagnostic capabilities (Gao et al., 2024). This approach not only enhances disease recognition but also provides a more comprehensive risk assessment for patients.

The implementation of AI in real-world healthcare settings is progressively transforming traditional paradigms of disease screening and management. In ophthalmic practice, AI technologies integrated with mobile devices and cloud-based platforms have enabled multi-scenario coverage extending from clinical facilities to household environments. For home-based applications, Chen et al. (2023) developed a smartphone application capable of detecting pediatric visual impairments through simplified visual acuity testing and image analysis, achieving an area under the receiver operating characteristic curve (AUROC) of 0.859 and facilitating early intervention (Chen et al., 2023). Similarly, Khurana et al. (2021) created a mobile platform that continuously monitors visual function changes in patients with diabetic retinopathy (DR) and age-related macular degeneration (AMD), enabling real-time data transmission to healthcare providers for effective chronic disease management (Khurana et al., 2021). In clinical settings, Heydon et al. (2021) demonstrated AI’s operational efficiency through a deep convolutional neural network (DCNN) trained on 128,000 fundus images. This system accomplished referral decision-making (“referable” vs. “non-referable”) within 15 s, exhibiting 94.7% sensitivity and 92.6% specificity. Notably, the implementation reduced manual review requirements by 83% and shortened screening cycle duration from 14 days to 2 days, substantially enhancing operational efficiency (Heydon et al., 2021). These case studies illustrate that AI technologies not only improve diagnostic accuracy and workflow efficiency but also extend medical services beyond conventional clinical environments through mobile-cloud integration. This dual capability enables broader population coverage and more efficient disease management across healthcare continua.

The cost-effectiveness advantages of AI in ophthalmic disease screening have been validated through multiple empirical studies, yet its sustainability requires systematic optimization. Research within the United Kingdom National Health Service (NHS) demonstrated that clinician-patient collaborative training mechanisms improved screening adherence rates by 19 percentage points, establishing a foundation for technology adoption (Willis et al., 2023). Lin et al. (2023) further revealed significant economies of scale in AI-based screening programs: when regional imaging data centers served populations exceeding five million, the per-case cost for diabetic retinopathy (DR) screening decreased from $58.7 to $36.4 (37.9% reduction) (Lin et al., 2023). In resource-limited settings, Xiao et al. (2021) innovated a mobile screening unit model that achieved 92.3% AI device utilization through pre-screening triage by community health workers, coupled with an 18.7% regional cost recovery effect (Xiao et al., 2021). However, two critical challenges emerged from current research: First, performance degradation of AI systems during longitudinal deployment was observed, as evidenced by Willis et al.'s finding of a 2.3 percentage point increase in missed diagnosis rates for atypical glaucoma after 18 months of implementation (Willis et al., 2023). Second, rigid constraints in human resource allocation were identified, where AI’s cost-effectiveness advantage becomes inverted when specialist review time exceeds 8 min per case. These findings underscore that developing sustainable AI screening systems necessitates a three-dimensional safeguard mechanism encompassing: 1) periodic system updates to maintain diagnostic performance, 2) tiered workforce training programs to optimize human-AI collaboration, and 3) real-time cost surveillance to ensure economic viability. Addressing these multidimensional requirements represents a crucial direction for future implementation research.

#### 2.2.2 Long term monitoring system

Long-term patient follow-up and disease monitoring are essential for effective disease management. Traditional follow-up methods rely on regular hospital visits and subjective doctor evaluations, which often result in high costs, low efficiency, and fragmented information (Long et al., 2020). AI-driven monitoring systems, leveraging machine learning and data mining, offer continuous, accurate, and efficient solutions, showing great potential in managing chronic diseases. For instance, Schmid et al. (2019) developed a mobile app for AMD self-monitoring, achieving high diagnostic accuracy (AUC: 0.799 for dry AMD, 0.969 for wet AMD), surpassing traditional methods (Schmid et al., 2019). For cataract monitoring, Long et al. created the CC-Guardian model, which uses Bayesian and deep learning for personalized care, including telehealth follow-ups. The model’s telehealth module demonstrated high accuracy, with a sensitivity of 0.959, specificity of 0.945, and AUC of 0.981, matching internal validation results (Long et al., 2020). Building effective AI models requires annotated clinical data for training and independent datasets for testing. Continuous monitoring and updates are crucial to maintaining their reliability, especially as new data and disease patterns emerge. This approach ensures that models remain accurate, generalizable, and capable of transforming the management of chronic eye diseases.

In summary, intelligent long-term monitoring systems have greatly improved chronic disease management by integrating diverse data and advanced algorithms. However, challenges remain: patient compliance and data quality, especially in elderly populations, can affect performance; long-term stability and update mechanisms need validation; and privacy and data security require urgent attention. The continuous optimization and refinement of intelligent long-term monitoring systems are poised to bring transformative changes to chronic disease management, driving the advancement of public health toward a more scientific and intelligent future.

### 2.3 Ophthalmic disease risk prediction and early warning

In recent years, with the continuous advancements in machine learning and big data analysis, researchers have developed various models to predict the occurrence of eye diseases. There have been many reviews that have reported the application in glaucoma, diabetic retinopathy, myopia, age-related macular degeneration and other diseases (Tan and Wong, 2022; Bullimore et al., 2023). These technologies automatically analyze medical images like fundus images and OCT scans, using large-scale clinical data (e.g., patient history, genetics, lifestyle, and ophthalmic exam results) and complex algorithms to identify and quantify risk factors for specific eye diseases. For example, Nugawela et al. developed and validated a risk model to predict vision-threatening diabetic retinopathy (DR) in patients with type 2 diabetes (Nugawela et al., 2022). By using extensive clinical data and retinal images, the model, built with machine learning algorithms, was tested in resource-limited environments. The results demonstrated high accuracy (C statistics ranging from 0.778 to 0.832) and practicality, effectively reducing resource consumption in DR screening while improving screening efficiency (Nugawela et al., 2022). Similarly, Li et al. proposed a deep learning system based on retinal images to predict the onset and progression of glaucoma (Li F. et al., 2022). By analyzing subtle features in retinal images, the system identifies potential glaucoma risks earlier than traditional methods. The results confirmed that the system performs well in predicting both the incidence and progression of glaucoma, achieving an AUROC of 0.90 (0.81–0.99) (Li F. et al., 2022). A summary of these models is in Table 3.

**TABLE 3:** Summary of algorithm/model for predicting the occurrence of ophthalmic diseases.

No.	Algorithm/Model	Application	References
1	Genome wide meta analysis prediction model	Identifies risk loci and combines genetic and clinical data to improve AMD prediction accuracy	He et al. (2024)
2	Cox proportional hazards model	Uses EHR data for early diagnosis and intervention of vision-threatening retinopathy	Nugawela et al. (2022)
3	DiagnosetNet algorithm	Predicts glaucoma from fundus photos, enhancing early detection and diagnosis accuracy	Li et al. (2022a)
4	FusionNet (Multimodal AI algorithm)	Integrates visual field and OCT data for improved prediction of glaucoma optic neuropathy	Xiong et al. (2022)
5	G-Risk (Risk regression model)	Deep learning model that screens glaucoma from fundus images, suitable for large-scale screening	Hemelings et al. (2023)
6	RESNET 101 based deep learning algorithm	Predicts myopia from fundus photos and integrates blockchain for data transparency and verification	Tan et al. (2021)
7	RAIDS (Retinal AI diagnosis system)	High accuracy and robustness across different diseases, ideal for comprehensive eye screening	Dong et al. (2022)

AI technology also has demonstrated significant potential in predicting the progression of ophthalmic diseases. By utilizing big data analysis and machine learning models, it provides ophthalmologists with more accurate prognosis assessments and disease management recommendations. Leveraging large-scale clinical and high-resolution image data, AI systems can identify early signs and key risk factors, enabling dynamic monitoring of conditions such as diabetic retinopathy (Yang et al., 2023), glaucoma (Dean et al., 2025), and myopia (Huang et al., 2023; Wang Y. et al., 2023). For diabetic retinopathy (DR) prediction, Bora et al. developed a deep learning-based risk prediction model using color fundus photos. In internal validation, the model achieved an area under the receiver operating characteristic curve (AUC) of 0.79 (95% CI 0.77–0.81), enabling early intervention and personalized management of DR in diabetic patients (Bora et al., 2021). Further advancing this, Dai et al. proposed a deep learning system that analyzes large-scale longitudinal datasets to predict the timeline of disease progression in DR patients (Dai et al., 2024). The system demonstrated a consistency index of 0.754–0.846 and a Brier score of 0.153–0.241 over 5 years, significantly enhancing prediction accuracy and reliability (Dai et al., 2024). For myopia prediction, Huang et al. introduced a time-aware multimodal deep learning model, which integrates data on axial length, diopter, and family history (Huang et al., 2023). The model achieved a precision with an error of just 0.103 diopters, well within clinically acceptable standards, offering a valuable tool for managing myopia in children and adolescents (Huang et al., 2023). Similarly, Wang et al. developed a machine learning model to predict the long-term vision outcomes of high myopia, providing crucial insights for patient follow-up and treatment (Wang Y. et al., 2023). In glaucoma management, Dean et al. verified a deep learning-based visual field prediction tool, which analyzes patient visual field data to forecast glaucoma progression (Dean et al., 2025). Ang et al. Used machine learning method to analyze the factors affecting the 10-year survival rate of grafts in patients with corneal endothelial disease. The results show that the machine learning model can accurately predict the success rate and long-term survival rate of transplantation by comprehensively analyzing the clinical characteristics of patients, transplantation methods and postoperative management (Ang et al., 2022). This finding is crucial for preoperative evaluation and personalized surgical planning, improving outcomes. A summary of these and other eye disease risk prediction models and algorithms is provided in Table 4.

**TABLE 4:** Summary of algorithm/model for predicting the development of ophthalmic diseases.

No.	Algorithm/Model	Application	References
1	AutoML model	Multimodal feature fusion using ultra-widefield imaging automatically detects early lesions (e.g., microaneurysms, exudates) and predicts 3-year progression risk (AUC 0.92).	Silva et al. (2024)
2	DenseNet-161 model	Quantitative OCTA parameters integrating macular vessel density, nonperfusion area, and foveal avascular zone morphology achieved 89% sensitivity in predicting	Yang et al. (2023)
3	Spatial ordered CNN model	Spatiotemporal modeling with 3D convolutional layers detects visual field changes, reducing false-positive alerts by 37% compared to linear regression model.	Shon et al. (2022)
4	DeepSeeNet model	Multimodal transfer learning with Inception-ResNet-v2 extracts drusen/pigmentary abnormalities from fundus photos, achieving a C-index of 0.85 for 5-year late AMD risk prediction.	Peng et al. (2020)
5	GLIM-Net model	Time-aware self-attention mechanisms process irregular optic disc images with positional encoding, showing 23% lower mean absolute error in predicting 5-year MD slope compared to LSTM baselines.	Hu et al. (2023)
6	Inception-v3 based deep learning model	Lesion-specific gradient-weighted class activation mapping (Grad-CAM) locates exudates/hemorrhages, achieving 93% sensitivity for predicting 5-year proliferative DR progression.	Bora et al. (2021)
7	Deepdr plus system	The Cox proportional hazards-deep learning hybrid model stratifies patients into rapid (1.2 years), intermediate (3.5 years), and slow (>5 years) progression groups using the DeepDR Score from fundus features.	Dai et al. (2024)
8	T-LSTM based deep learning model	Time-gated covariate weighting combines refractive error, axial length, and optic disc tilt, achieving AUC 0.88 for predicting ≥1D myopia progression within 2 years.	Huang et al. (2023)
9	Machine learning model for long-term vision prediction	Multivariable risk stratification incorporating posterior staphyloma severity, choroidal thickness, and ERG amplitude demonstrated C-index 0.79 for 5-year risk of ≥0.2 logMAR visual acuity decline in high myopia.	Wang et al. (2023b)
10	Kalman filter-based machine learning model	Adaptive process noise calibration in state-space modeling improved 5-year glaucoma progression prediction accuracy to 89% in the United Kingdom Glaucoma Cohort, with cross-center consistency ICC >0.85.	Dean et al. (2025)

AI-driven predictive models demonstrate theoretical potential for optimizing public health strategies in ophthalmology, particularly in risk stratification and preventive intervention design. While the integration of algorithmic systems with clinical knowledge could theoretically enhance personalized treatment, this synergy may be compromised by the black-box nature of deep learning outputs. Furthermore, overreliance on predictive analytics risks generating medical and ethical concerns. Sustainable implementation requires the integration of AI’s computational capabilities with clinicians’ domain expertise to achieve more precise, equitable, and explainable clinical decision-making.

### 2.4 Optimal allocation of ophthalmic medical resources

Telemedicine, as a crucial application of AI in ophthalmic public health, has significantly enhanced the accessibility and efficiency of eye care services, particularly in remote areas and resource-constrained primary healthcare institutions. AI-assisted remote diagnosis, treatment planning, and consultation systems have demonstrated substantial improvements in service delivery models (Taylor et al., 2007). By overcoming geographical barriers, telemedicine has emerged as a pivotal strategy for addressing the unequal distribution of medical resources. Its current operational paradigm predominantly follows an asynchronous consultation system characterized by “front-end portable device data acquisition and cloud-based expert diagnosis.” For instance, Liu et al. evaluated the application of automated imaging optical coherence tomography (OCT) devices in remote diagnosis and monitoring of retinal diseases. This technology reduces reliance on specialized technicians while markedly improving service convenience and accessibility in resource-limited regions (Liu Z. et al., 2024). Concurrently, the proliferation of smartphone technology has enabled novel approaches in ophthalmic examinations. Vilela et al. investigated the integration of AI-driven smartphone platforms for high-precision retinal image analysis (Vilela et al., 2024). The portability and user-friendly interface of these devices make them ideal tools for ophthalmic screening in remote areas, where patients can perform self-examinations after minimal training, thereby further enhancing service accessibility and operational convenience. The synergistic combination of automated imaging OCT devices with AI-enhanced smartphone platforms has significantly expanded both the coverage and efficiency of ophthalmic public health services. This technological integration enables broader populations to receive timely, high-quality diagnostic evaluations and longitudinal monitoring, representing a paradigm shift in equitable healthcare delivery.

In the field of AI-assisted telemedicine, Chen et al. emphasized the application of 5G technology in real-time remote retinal laser photocoagulation therapy, which significantly enhanced the accuracy and safety of teleoperated surgical procedures. Furthermore, microsurgical robotics is emerging as a cutting-edge advancement in remote ophthalmic treatment (Chen et al., 2021). Ladha et al. demonstrated the superiority of robot-assisted techniques over conventional manual methods through simulated subretinal injection experiments, showing that enhanced injection precision and reduced human error rates substantially improved both therapeutic outcomes and patient safety in gene therapy applications (Ladha et al., 2023). In cataract surgery, Garcia Nespolo et al. evaluated AI-guided tools for phacoemulsification procedures, revealing that real-time provision of critical parameters enabled better surgical control, reduced complications, and improved operational safety (Garcia Nespolo et al., 2022).

However, the equity and accessibility of ophthalmic telemedicine still require further improvement. For example, in certain regions of India, community health workers use portable fundus cameras (such as the validated Peek Retina) adapted for smartphones to capture retinal images of patients, which are then securely transmitted to regional medical centers for remote assessment and referral by ophthalmologists (Wintergerst et al., 2020). This model has significantly increased screening coverage in resource-poor areas, but its daily processing capacity is typically limited to fewer than 200 cases due to the inefficiency of manual image reading, making it challenging to meet the demands of large-scale screening. A more advanced integration of AI and telemedicine provides a breakthrough solution to this issue. By deploying lightweight AI models on the device side, it is possible to provide instant feedback on image quality and preliminary diagnosis, with only high-risk cases being uploaded to the cloud for expert review. This approach has been proven feasible in pilot projects in some African countries, where screening throughput has been increased to over 1,000 cases per day, while maintaining a clinically acceptable error and missed diagnosis rate (Cleland et al., 2016; Rajkomar et al., 2019). To further promote and optimize ophthalmic telemedicine, a range of comprehensive measures is needed, including improving the accessibility of digital devices, expanding the virtual services of ophthalmic hospitals, enhancing the digital literacy of primary healthcare workers, and optimizing communication methods for telemedicine, thus building a more efficient and inclusive eye care system.

### 2.5 Ophthalmology health education

AI technology offers a new learning pathway for primary healthcare staff, significantly enhancing their professional skills and service quality. AI-powered online education platforms can deliver tailored training courses based on the specific needs and knowledge levels of individual healthcare workers (Fang et al., 2022). These platforms also update content in real-time, ensuring that medical personnel stay current with the latest advancements in medical knowledge, diagnostics, and treatment guidelines (Wu et al., 2020). Additionally, AI integrates virtual reality (VR) and augmented reality (AR) to simulate real-life eye surgeries, diagnostic, and treatment scenarios, providing immersive training experiences for primary care providers (Ong et al., 2021; Ma et al., 2022). This method not only boosts engagement and participation but also helps improve clinical skills (Popov et al., 2024). By offering opportunities for repeated practice and instant feedback, medical staff gain greater confidence and precision in handling various eye health issues (Bakshi et al., 2021). Moreover, AI-assisted remote consultation systems enhance communication between primary care workers and specialists, enabling real-time transmission of patient records and images, and facilitating multi-party video consultations. This allows frontline workers to engage in detailed discussions with experts, gaining valuable guidance. Through its support in remote diagnosis, consultations, and training, AI has significantly improved the accessibility and quality of medical services, addressing the issue of uneven distribution of healthcare resources.

In addition, AI also contributes to the popularization of patient health science. With the continuous maturity and popularization of AI technology, developers have used the AI platform to create more health science popularization applications, which can provide personalized eye health education content according to the specific situation of patients or the receiving population. For example, for patients with diabetes, the AI system can push knowledge about the prevention, early detection and treatment of diabetic retinopathy, help patients better understand their disease, and take effective self-management measures (Wu et al., 2023). For doctors, AI can simulate the operation process and help doctors better understand the difficult operations, such as cataract surgery (Ament and Henderson, 2011). The health education capabilities of AI are not limited to the mere dissemination of text and images. By NLP technology, AI can understand patients’ verbal inquiries and provide more direct and personalized health advice through conversational interactions (Wang et al., 2020). This function is especially suitable for middle-aged and elderly people who are not familiar with Internet operation, reducing the threshold for them to acquire eye health knowledge. The development and application of these technologies have laid a solid foundation for achieving the goal of eye health for all, and demonstrated their great potential and prospects in the field of medical education. Through AI technology, it can not only improve the professional skills and service quality of grass-roots medical personnel, but also enable patients to obtain professional and instant health information in daily life, enhance patients’ coordination and compliance with disease treatment, and further promote the optimization and efficiency of public health services.

## 3 Challenges and prospects

### 3.1 The interoperability problem in AI systems

The systemic interoperability challenges in ophthalmic clinical practice remain a critical barrier to AI-EHR (Electronic Health Record) integration, rooted in the inherent heterogeneity of healthcare infrastructure, particularly the diversity of data storage modalities (Kirshner et al., 2021). The predominant storage of medical data in unstructured formats imposes significant computational burdens during AI preprocessing, necessitating standardized contextualization to ensure data consistency (Wang et al., 2018). While existing interoperability standards like HL7 FHIR® prove effective in general medical domains, their application to ophthalmic imaging modalities (e.g., OCT and fundus photography) reveals deficiencies in metadata standardization frameworks, failing to meet AI models’ requirements for structured data inputs (Finkelstein et al., 2024). To address these challenges, the development of intelligent adaptation systems emerges as a priority solution. These systems employ dynamic semantic alignment technologies to reconcile heterogeneous data formats, enabling seamless integration of AI models into clinical workflows without requiring large-scale system overhauls. Empirical evidence demonstrates that this approach reduces AI deployment timelines by over 50% while enhancing clinical operational efficiency (Reddy, 2024). However, persistent technical fragmentation among medical device manufacturers and commercial competition continue to hinder large-scale implementation. Consequently, policy-level coordination is imperative to establish ophthalmology-specific imaging data interoperability protocols. Such initiatives should focus on unifying industry standards through multistakeholder consensus, thereby facilitating the effective adoption and integration of AI technologies in ophthalmic practice. Regulatory bodies must prioritize the creation of specialty-adapted validation frameworks that balance technical feasibility with clinical utility, ensuring both algorithmic performance and workflow compatibility.

### 3.2 Data security and privacy

The application of AI to EHRs and medical image processing, while enhancing healthcare efficiency and diagnostic accuracy, poses significant data privacy and security challenges. These challenges primarily stem from the inherent complexity of medical data, the necessity for cross-institutional data sharing, intrinsic vulnerabilities in AI models, and evolving regulatory frameworks (Daneshvar et al., 2024). A multi-layered and comprehensive strategy is required to address these concerns. Firstly, data must undergo rigorous anonymization and de-identification processes, including the removal or substitution of personal identifiers. Advanced privacy-preserving techniques such as differential privacy and federated learning should be implemented to safeguard sensitive information during AI model training (Sung et al., 2021). Secondly, robust data encryption protocols combined with strict access control mechanisms are essential to prevent unauthorized access or tampering during data transmission and storage (Huang et al., 2022). From a technical perspective, enhancing AI model security through secure multi-party computation and homomorphic encryption can mitigate risks of adversarial exploitation (Gong et al., 2020). Comprehensive model testing and validation procedures must be mandated to identify potential vulnerabilities. Furthermore, establishing a robust data governance framework is critical to ensure compliance with international regulations including HIPAA and GDPR (Khatri and Brown, 2010; Rumbold and Pierscionek, 2017). This should be supplemented by periodic security audits and systematic risk assessments to proactively detect and remediate potential breaches. By implementing these integrated measures, the privacy and security risks associated with AI-driven medical data processing can be substantially mitigated, thereby better protecting patient rights while maintaining the transformative potential of AI in healthcare. Future research directions should emphasize the development of standardized evaluation metrics for privacy-preserving technologies and the creation of harmonized regulatory guidelines across jurisdictions.

### 3.3 Data quality and bias

The clinical application of AI in ophthalmology faces significant challenges in data quality, particularly regarding incompleteness, inaccuracy, and bias, which must be systematically addressed to ensure AI system reliability and safety. Data incompleteness, manifested through incomplete medical records or missing diagnostic results, directly compromises the integrity of AI model training and diagnostic accuracy (Rajpurkar et al., 2022). To address this, standardized data collection protocols must be established, supplemented by automated tools for data completion and statistical approaches such as imputation techniques for missing values (Shi et al., 2021). Data inaccuracy arising from entry errors, measurement deviations, or equipment malfunctions necessitates rigorous data validation and cleansing strategies. This includes implementing cross-validation with multi-source data and employing outlier detection techniques to ensure data authenticity (Cai and Zhu, 2015). The most complex challenge lies in data bias, which may stem from sample selection bias, ethnic disparities, or geographical variations, potentially leading to suboptimal model performance in specific populations (Zech et al., 2018). For instance, choroidal melanocytic variations in African populations risk algorithmic misclassification as pathological features (Boutros et al., 2024), while retinal pigment epithelial subdeposits morphology prevalent in Caucasian populations presents overgeneralization risks in Asian datasets (Bressler SB et al., 2007). These examples underscore the critical importance of data diversity and algorithmic fairness. Mitigation strategies should focus on constructing diverse, representative datasets encompassing heterogeneous population characteristics. Data augmentation techniques could be employed to synthesize underrepresented population data, coupled with the integration of bias detection and correction mechanisms during model training (Zhang et al., 2018). Through comprehensive improvements in ophthalmic AI data quality, we can ultimately develop trustworthy AI diagnostic systems that meet rigorous clinical standards. This multidimensional approach ensures equitable performance across diverse populations while maintaining diagnostic precision and operational safety.

### 3.4 Algorithmic transparency and explainability

Addressing the “black box” challenge in ophthalmic AI - enhancing the transparency and interpretability of AI algorithms - represents a pivotal obstacle in advancing clinical translation (Li et al., 2024). While deep learning models demonstrate exceptional performance in image recognition tasks, their intricate decision-making processes often hinder clinicians’ comprehension and trust (Topol, 2019). The development of AI systems capable of generating natural language explanations proves essential for enabling intuitive understanding of algorithmic reasoning among medical professionals. Concurrently, explicit quantification of uncertainty in algorithm outputs could further augment clinical decision-making by contextualizing predictions within probabilistic frameworks (Chua et al., 2022). A retrospective analysis revealed that many oversimplified explanation interfaces inadvertently justified hypersensitivity to imaging artifacts through spurious pathophysiological correlations (Ong Ly et al., 2024). This underscores the necessity for sophisticated explanation architectures, such as dual-layer interpretability frameworks. At the technical stratum, these systems should provide granular operational details including feature importance metrics and model confidence levels. The clinical stratum must translate these technical parameters into medically relevant concepts, synthesizing patient history, symptomatology, and imaging characteristics into explanations aligned with clinical reasoning paradigms. An interactive interface enabling dynamic adjustment of explanation specificity based on clinician feedback could optimize human-AI collaboration. By aligning algorithmic outputs with clinicians’ cognitive schemata through context-aware explanations, such systems may enhance perceived trustworthiness and practical utility (Holzinger et al., 2019). Future implementations should prioritize bidirectional communication channels that adapt explanatory depth to clinical context and operator expertise, thereby fostering responsible integration of AI in ophthalmic practice.

### 3.5 Ethics and regulation

The integration of AI into ophthalmic public health systems has precipitated critical ethical and regulatory challenges that demand rigorous consideration and systematic responses. Conventional medical device certification protocols mandate fixed algorithm versions, yet real-world AI implementations necessitate continuous learning from evolving data streams. The degradation of AI model performance following clinical deployment, often attributed to data distribution shifts such as variations in imaging equipment parameters or evolving patient demographics, has emerged as a critical concern in medical AI implementation. Empirical evidence indicates that algorithm updates lacking rigorous validation against novel environmental data distributions - particularly when over-adapted to institution-specific imaging acquisition protocols - may precipitate systematic diagnostic inaccuracies. This phenomenon has been identified as a central challenge in the real-world deployment of medical AI systems, underscoring the imperative for robust cross-institutional validation frameworks to ensure algorithmic generalizability across heterogeneous clinical environments (Finlayson SG, 2021). This has catalyzed the exploration of dynamic certification mechanisms permitting constrained algorithm iterations within predefined confidence intervals, coupled with real-time performance monitoring systems. Diagnostic errors in medical AI may originate from data anomalies, model deficiencies, or clinical implementation variances, rendering conventional unitary liability models inadequate (Wiens et al., 2019). A stratified accountability framework is therefore imperative to delineate distinct responsibilities among developers, healthcare providers, and regulatory bodies. Crucially, AI systems should be positioned as clinical decision-support tools rather than autonomous diagnosticians (Pinsky et al., 2022). Establishing a bidirectional calibration-based interactive paradigm enables AI to deliver data-driven recommendations while clinicians contextualize outputs with experiential knowledge, thereby facilitating precise error attribution and dynamic risk mitigation in technologically embedded clinical settings (Rajkomar et al., 2019). This human-AI collaborative framework addresses the practical needs of technology applications but also ensures patient welfare and sustainable healthcare ecosystems through shared accountability and value alignment.

## 4 Conclusion

This paper provides a comprehensive analysis of the application and value of AI in ophthalmic public health, with a particular focus on its transformative impact on screening, diagnosis, monitoring, continuing medical education, and telemedicine. As AI technology continues to evolve, innovative solutions are increasingly being integrated into ophthalmology, helping to improve early disease detection, enhance diagnostic accuracy, monitor patient health in real time, elevate the professionalism of healthcare providers, and optimize the distribution of medical resources. While current implementations demonstrate measurable success in augmenting detection sensitivity and optimizing clinical workflows, critical research gaps demand prioritized investigation, including addressing interoperability challenges stemming from heterogeneous data formats, enhancing data security through anonymization and federated learning, developing explainable AI systems to resolve the “black box” dilemma, mitigating data quality and bias via standardized collection and validation protocols, and establishing dynamic ethical and regulatory frameworks to harmonize innovation with patient safety. We believe that AI in ophthalmology will realize a future where every person can benefit from superior eye care, regardless of their circumstances.
